# Raised intrathecal levels of APRIL and BAFF in patients with systemic lupus erythematosus: relationship to neuropsychiatric symptoms

**DOI:** 10.1186/ar2484

**Published:** 2008-08-22

**Authors:** Annie George-Chandy, Estelle Trysberg, Kristina Eriksson

**Affiliations:** 1 Department of Rheumatology and Inflammation Research, Guldhedsgatan 10A, 413 46 Gothenburg, Sweden; 2 Department of Infection Immunology, Statens Serum Institut, Artillerivej 5, 232 300 Copenhagen, Denmark; 3 Rheumatogy Unit at Karolinska University Hospital/Huddinge, Hälsovägen 141, 141 52 Huddinge, Sweden

## Abstract

**Introduction:**

The tumour necrosis factor (TNF) family ligands BAFF (B-cell activating factor of TNF family) and APRIL (a proliferation-inducing ligand) are essential for B-cell survival and function. Elevated serum levels of BAFF and APRIL have been reported earlier in patients with systemic lupus erythematosus (SLE). Since autoantibody formation in the central nervous system (CNS) is a distinct feature of neuropsychiatric SLE (NPSLE), we have investigated whether NPSLE is associated with an enhanced intrathecal production of APRIL and BAFF.

**Methods:**

Levels of BAFF and APRIL in cerebrospinal fluid (CSF) and serum from healthy controls, SLE patients without CNS involvement, and patients with NPSLE were determined by enzyme-linked immunosorbent assay. Interleukin-6 (IL-6) levels were determined by an IL-6-specific bioassay.

**Results:**

SLE patients had levels of APRIL in CSF that were more than 20-fold higher and levels of BAFF in CSF that were more than 200-fold higher than those of healthy controls. Separate analyses of SLE patients with and without CNS involvement revealed that NPSLE patients had enhanced levels of APRIL in CSF. BAFF and APRIL were likely produced locally in the CNS as CSF and serum levels did not correlate. Moreover, CSF levels of APRIL correlated with BAFF but not with IL-6, suggesting that APRIL and BAFF in the CNS are regulated together but that they are produced independently of IL-6.

**Conclusion:**

To our knowledge this is the first study to show elevated levels of BAFF and APRIL in CSF of SLE patients. APRIL was augmented in NPSLE patients compared with SLE patients without CNS involvement. APRIL and BAFF antagonists breeching the blood-brain barrier therefore could have beneficial effects on SLE patients, in particular patients with NPSLE.

## Introduction

Systemic lupus erythematosus (SLE) is a chronic, usually life-long, potentially fatal autoimmune disease characterised by an increased production of autoantibodies, impairment of B- and T-cell functions, cytokine production, and immune complex deposition. SLE is manifested, for example, in neurological, dermal, haematological, musculoskeletal, and renal symptoms [1]. Central nervous system (CNS) involvement has been reported to occur in 14% to 75% of patients with SLE and is a major factor contributing to morbidity and mortality in patients [2]. The aetiology of neuropsychiatric SLE (NPSLE) includes autoantibody production specific for brain structures, immune complex depositions, microangiopathy, and intrathecal production of proinflammatory cytokines. Seizures, stroke, depression, psychoses, and disordered mentions are manifestations of this disease [3]. Beneficial treatment in the form of cytotoxic drugs is available [4] but requires early recognition of CNS involvement. Due to the multiple pathogenic mechanisms causing NPSLE, there is no single confirmatory diagnostic test for NPSLE. Several clinical, laboratory, and radiographic test findings are reported to be abnormal in some but not all patients. Magnetic resonance imaging (MRI) of the brain has been shown to be valuable in detecting even minor NPSLE-induced lesions [5]. Pleocytosis and elevated protein levels are found in some but not all NPSLE patients. Elevated concentrations of IgG in cerebrospinal fluid (CSF) IgG-albumin ratio, IgG index, and the presence of oligoclonal bands have all been described with varying frequencies. Increased levels of interleukin (IL)-1 [6], IL-6 [6,7], IL-8 [8], and interferon-gamma (IFN-γ) [9] have been found in CSF of NPSLE patients. We have reported earlier that patients with NPSLE displayed elevated CSF levels of matrix metalloprotease-9 well as intrathecal neurofilament (NFL) and glial fibrillary acidic protein (GFP) [10], which are markers for neuronal and astroglial brain damage.

The tumour necrosis factor (TNF) family ligands BAFF (B-cell activating factor of TNF family) and APRIL (a proliferation-inducing ligand) are implicated in several immunological phenomena such as peripheral B-cell survival, CD40L-independent antibody production and isotype switching, autoimmunity as well as tumour cell growth [11,12]. BAFF is expressed on the cell surface or cleaved and secreted [12], while APRIL is cleaved from the Golgi and solely exists as a secreted soluble ligand [13]. BAFF and APRIL share two receptors: B-cell maturation antigen (BCMA) and transmembrane activator and calcium-modulating cyclophilin ligand interactor (TACI), which are found mainly on B cells and plasma cells [14]. In addition, BAFF binds to BAFF receptor found mainly on B cells, plasma cells, and some subsets of T cells [15,16], while APRIL interacts with heparin sulfate proteoglycans, which likely constitutes a third receptor for APRIL [17].

In the context of autoimmunity, both BAFF and APRIL are implicated in the establishment and/or maintenance of autoimmune disease. Abnormal serum levels of BAFF and APRIL have been observed in patients with rheumatoid arthritis [18], Sjögren syndrome [19], and SLE [20]. In SLE patients, increased serum levels of BAFF, APRIL, and BAFF/APRIL heterotrimers correlate with anti-double-stranded DNA autoantibodies and disease activity [21]. Gene polymorphism of APRIL has been reported to be associated with SLE [22]. The association between enhanced levels of BAFF and autoimmune disease in humans has been substantiated in mice rendered transgenic or deficient for this cytokine. Mice overexpressing BAFF develop a lupus-like phenotype characterised by high titres of anti-DNA antibodies, hypergammaglobulinaemia, and glomerulonephritis [23], while mice lacking BAFF are deficient in mature B cells and marginal zone B cells [24]. The association between elevated circulating levels of BAFF and polyclonal hypergammaglobulinaemia extends to humans as well. Increased serum and/or plasma levels of BAFF have been documented in SLE, rheumatoid arthritis, and Sjögren syndrome [25-27], all conditions associated with polyclonal hypergammaglobulinaemia. Overexpression of APRIL, in contrast, has not been associated with autoimmunity in mice but leads to enhanced IgM production, T-cell-independent type 2 humoral responses, and T-cell survival [28], while lack of APRIL is associated with enhanced numbers of effector/memory T cells and impaired IgA responses [29,30]. However, APRIL and BAFF/APRIL heterotrimers have been found to be elevated in sera and target organs of autoimmune disease patients, including SLE, Sjögren syndrome, multiple sclerosis (MS), and myasthenia gravis [31-34]. The purpose of this study was to examine BAFF and APRIL levels in the CSF of SLE patients with or without NPSLE in order to investigate whether BAFF and/or APRIL could have a role in the pathology of NPSLE.

## Materials and methods

### Participants

Seventy-nine patients who fulfilled at least four of the American Rheumatism Association (ARA) 1987 revised criteria for the classification of SLE [35] and who ranged in age from 19 to 75 years (mean age ± standard deviation [SD]: 45 ± 15 years; 66 females and 13 males mostly of Caucasian origin) were included in the study. All subjects were patients at the Department of Rheumatolgy, Sahlgrenska University Hospital. The patients were consecutively incorporated into the study. The disease duration varied between 0 and 41 years with a mean of 9 ± 9 years. The patients underwent a thorough clinical examination by an experienced rheumatologist and a neurologist. Examination of CNS signs and symptoms included lumbar puncture, neuropsychological tests, and MRI of the brain. The proposed definition of CNS lupus according to ARA criteria for SLE [35] appears inadequate, given that only two elements, psychosis and seizures, are included. We defined NPSLE as the presence of at least two of the following seven items occurring in association with clinical evidence of disease progression: (a) recent-onset psychosis, (b) transverse myelitis, (c) aseptic meningitis, (d) seizures, (e) pathological brain MRI, (f) severely abnormal neuropsychiatric tests [36], and (g) oligoclonal IgG bands in the CSF. The pathogenesis of anti-phospholipid-antibody-mediated brain damage is thrombotic rather than inflammatory, and it has previously been shown that stroke gives rise to increased values of GFP and NFL. Consequently, we decided to exclude this condition from the definition of CNS lupus. Patients with non-SLE causes of neurological events (for example, cerebral infections) were also excluded. Based on the criteria above, the patients were divided into two distinct groups: (a) patients with NPSLE (n = 37) and (b) patients with SLE but without any signs of NPSLE (n = 45). Clinical CNS and peripheral nervous system symptoms of included SLE patients are specified in Table 1. CSF from healthy blood donors with no previous history of neurological disorder and with a normal neurological status served as controls in the present study. The age of the control subjects (11 males and 9 females) was 38 ± 11 years. The Medical Ethics Committee at Göteborg University approved the study, and informed consent was obtained from all patients and participating blood donor volunteers after written and verbal information had been given.

**Table 1:** Clinical central nervous system/peripheral nervous system manifestations in systemic lupus erythematosus patients included in the study

Central nervous system manifestations	No NPSLE	NPSLE
Acute confusional state	0	2
Anxiety disorder	0	1
Aseptic meningitis	0	1
Cerebrovascular disease	9	6
Cognitive dysfunction	10	4
Demyelinating syndrome	2	4
Headache	9	3
Mood disorders	7	3
Movement disorder	0	0
Myelopathy	0	1
Psychosis	0	7
Fatigue	3	0
Mononeuropathy	1	0
Polyneuropathy	1	0

Each patient may have had multiple clinical manifestations of central nervous system involvement. NPSLE, neuropsychiatric systemic lupus erythematosus.

### Enzyme-linked immunosorbent assay measurements for BAFF and APRIL

CSF and serum samples were assayed for APRIL and BAFF by antigen-capture enzyme-linked immunosorbent assays (ELISAs). For the detection of human APRIL, a kit from Bender MedSystems (Vienna, Austria) was used according to the manufacturer's instructions. For the measurement of BAFF, ELISA plates were coated overnight with monoclonal mouse IgG1 anti-human BAFF (clone 137314; R&D Systems, Abingdon, Oxfordshire, UK) at 1 μg/mL in phosphate-buffered saline. After nonspecific binding had been blocked with 0.5% bovine serum albumin, the samples were added, followed by the detection antibody, biotinylated goat anti-human BAFF (R&D Systems). Streptavidin horseradish peroxidase and 3,3',5,5'-tetramethylbenzidine (Sigma-Aldrich, St. Louis, MO, USA) were used for detection. The reaction was stopped with 0.5 M H_2_SO_4_, and the enzyme activity was read at an optical density of 450 nm. A seven-point standard curve starting at 20,000 pg/mL of recombinant BAFF was generated. The recombinant human BAFF used for the standard curve was expressed in the mouse myeloma cell line NSO (R&D Systems).

### Interleukin-6 measurement

The cell line B 13.29, which is dependent on IL-6 for growth, has been described previously. For IL-6 determinations, the more sensitive subclone B9 was used [37,38]. B9 cells were harvested from tissue culture flasks, seeded into microtitre plates (Nunc, Roskilde, Denmark) at a concentration of 5,000 cells per well, and cultured in Iscove's medium supplemented with 5 × 10^5 ^mol/L 2-mercaptoethanol, 5% foetal calf serum (Sera Laboratories International, Haywards Heath, West Sussex, UK), penicillin (100 U/L), and streptomycin (100 μg/mL). CSF or serum samples were then added. (H3)thymidine was added after 68 hours of culturing, and the cells were harvested 4 hours later. The samples were tested in two-fold dilutions and compared with a recombinant human IL-6 standard (Genzyme, Cambridge, MA, USA). B9 cells were previously shown not to react with several recombinant cytokines, including IL-1α, IL-1β, IL-2, IL-3, IL-5, granulocyte-macrophage colony-stimulating factor, TNF-α, and IFN-γ. There was only a weak reactivity with purified monoclonal antibody specific for human IL-6 (Genzyme) in a neutralisation assay. Preincubation of 10 μg/mL of this antibody with either recombinant IL-6 or CSF containing naturally produced IL-6 (1 hour at 37°C) reduced proliferative responses of B9 indicator cells by an average of greater than or equal to 95% [39].

### Statistical analyses

All statistical analyses were performed using the GraphPad Prism software (GraphPad Software, Inc., San Diego, CA, USA). Nonparametric testing was performed by the Mann-Whitney test for comparison of two groups. Correlation was determined by Spearman correlation.

## Results

### Raised APRIL and BAFF levels in cerebrospinal fluid of systemic lupus erythematosus patients

**Figure 1:**
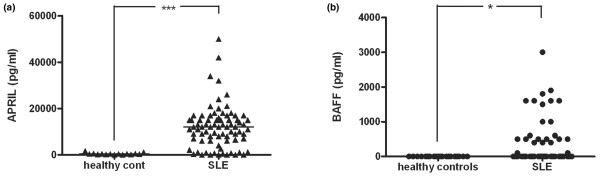
Raised APRIL and BAFF levels in cerebrospinal fluid of systemic lupus erythematosus (SLE) patients. Cerebrospinal fluid levels of **(a) **APRIL and **(b) **BAFF in SLE patients and in healthy controls. APRIL and BAFF levels are expressed in picograms per millilitre. Median values are depicted with a line. *P < 0.05, ***P < 0.001. APRIL, a proliferation-inducing ligand; BAFF, B-cell activating factor of tumour necrosis factor family.

We measured APRIL levels in the CSF of 79 SLE patients and 15 healthy controls. Intrathecal levels of APRIL were increased 24 times in SLE patients compared with healthy controls (mean ± SD of 10,835 ± 8,462 versus 455 ± 436 pg/mL; Figure 1a). No subjects in the group of healthy controls displayed APRIL levels over 1,700 pg/mL in CSF. BAFF levels in CSF were measured in 76 SLE patients and 20 healthy controls. Intrathecal levels of BAFF were significantly raised in SLE patients (216 ± 609 pg/mL) and were below detection levels in all healthy controls (Figure 1b).

### Raised APRIL levels in NPSLE patients compared with systemic lupus erythematosus patients without central nervous system symptoms

**Figure 2:**
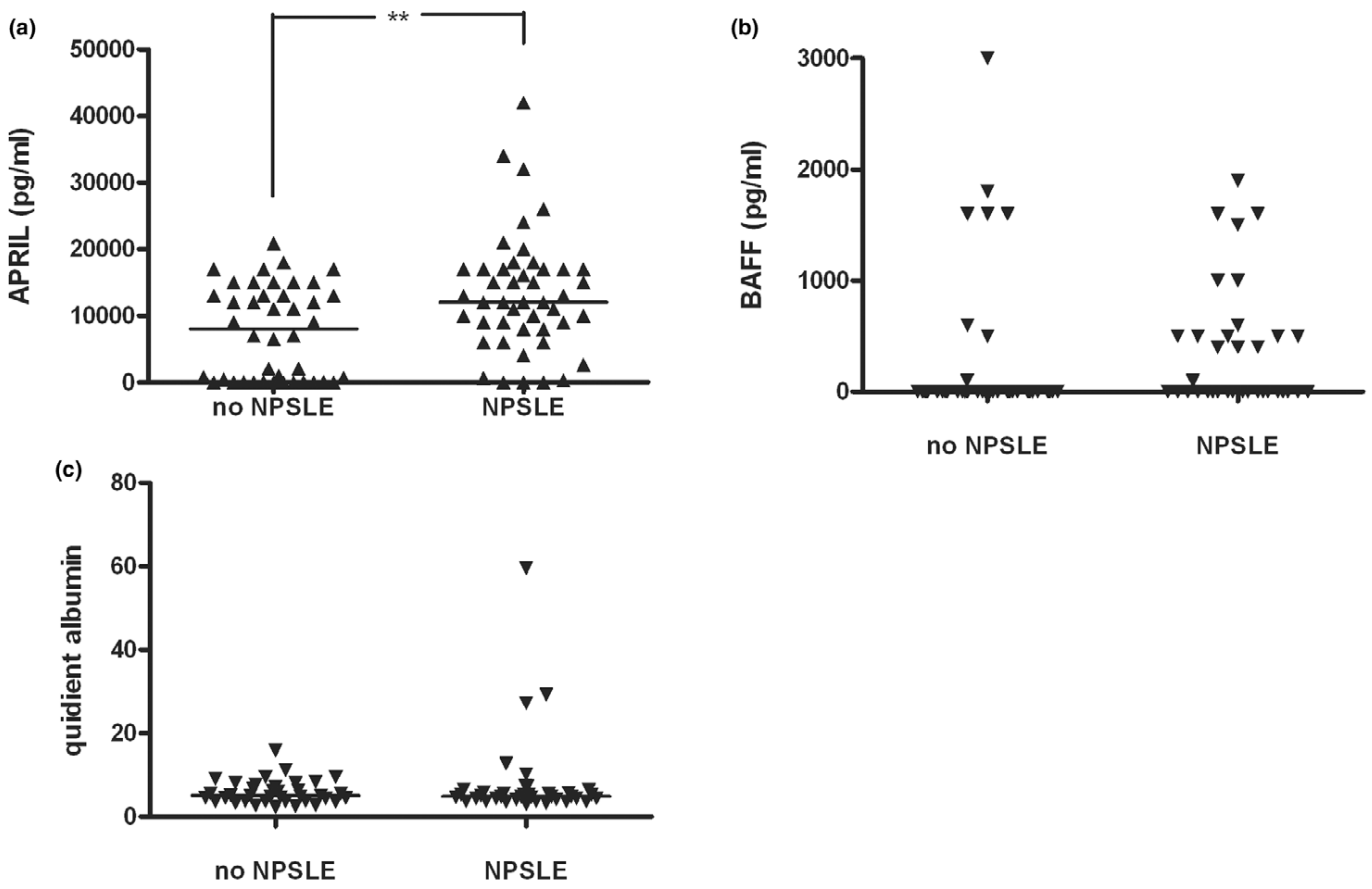
Raised APRIL levels in NPSLE patients compared with SLE patients without central nervous system symptoms. Cerebrospinal fluid (CSF) levels of **(a) **APRIL and **(b) **BAFF in SLE patients separated into cerebrally healthy SLE patients and patients with NPSLE. **(c) **The quotient of CSF albumin × 10^3^/serum albumin. Median values are depicted with a line. **P < 0.01. APRIL, a proliferation-inducing ligand; BAFF, B-cell activating factor of tumour necrosis factor family; NPSLE, neuropsychiatric systemic lupus erythematosus; SLE, systemic lupus erythematosus.

Separate analyses of APRIL levels in SLE patients with NPSLE and patients without CNS disease revealed that NPSLE patients had 1.5 times higher APRIL levels in CSF compared with SLE patients without overt CNS disease (13,677 ± 9,725 versus 8,672 ± 9,725 pg/mL; Figure 2a). No significant differences in BAFF levels could be detected in SLE patients with NPSLE compared with those without CNS disease (Figure 2b). As an indicator of blood-brain barrier function, the quotient of CSF albumin × 10^3^/serum albumin was analysed (normal value less than 6.5 to 8.0). No significant differences in albumin quotient could be detected between SLE patients with and without NPSLE (Figure 2c).

### No correlation between cerebrospinal fluid and serum levels of BAFF/APRIL

**Figure 3:**
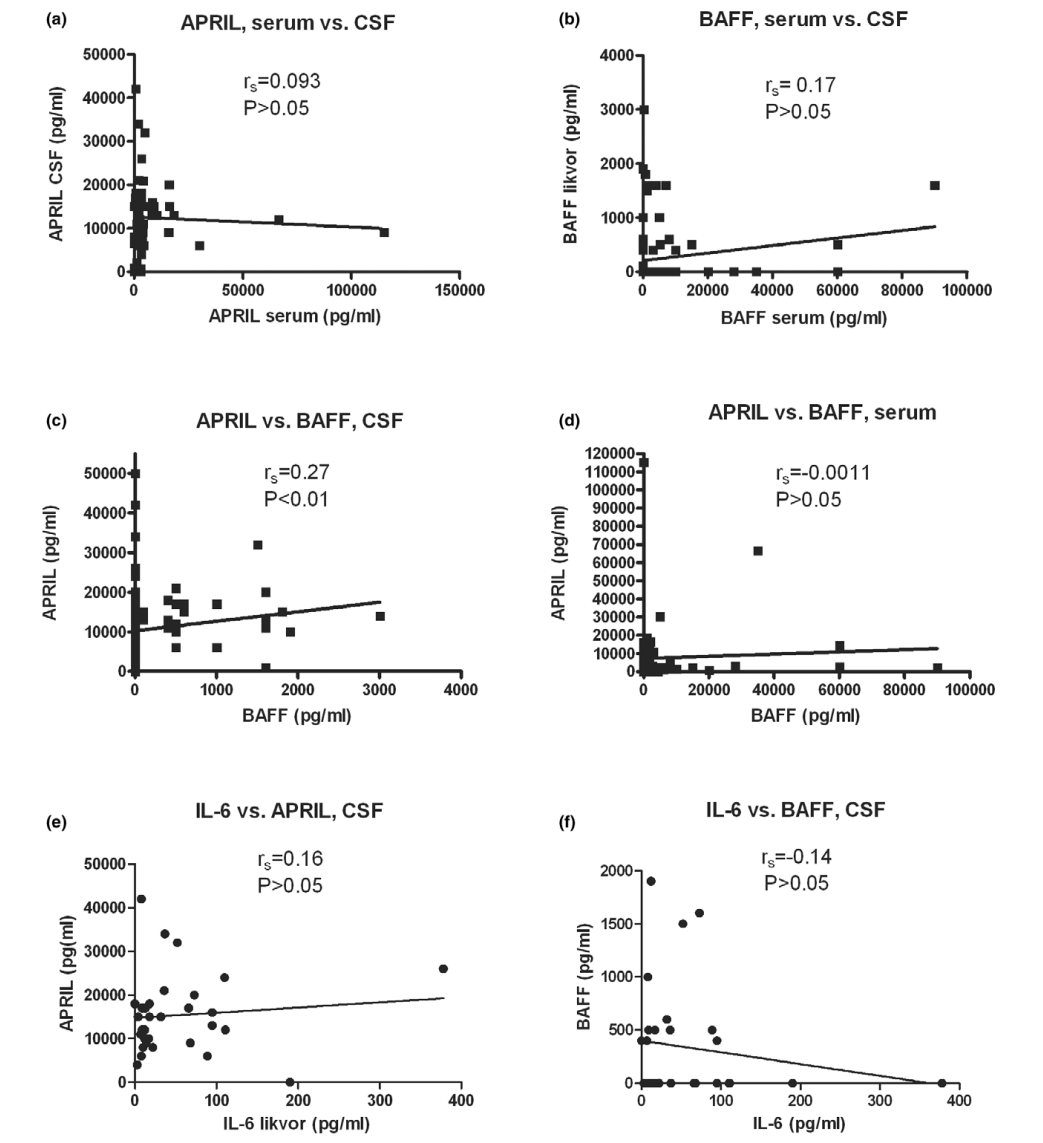
Correlation analyses of APRIL, BAFF, and IL-6. Correlation between levels of **(a) **APRIL in cerebrospinal fluid (CSF) and serum, **(b) **BAFF in CSF and serum, **(c) **APRIL and BAFF in CSF of systemic lupus erythematosus (SLE) patients, **(d) **APRIL and BAFF in serum of SLE patients, **(e) **IL-6 and APRIL in CSF of SLE patients, and **(f) **IL-6 and BAFF in CSF of SLE patients. APRIL, a proliferation-inducing ligand; BAFF, B-cell activating factor of tumour necrosis factor family; IL-6, interleukin-6.

The correlations between CSF and serum levels of APRIL and between CSF and serum levels of BAFF were analysed on 70 and 79 SLE patients, respectively. No correlation could be seen between APRIL levels in CSF and serum (Figure 3a), nor could any correlation be detected between BAFF levels in CSF and serum (Figure 3b).

### Correlation between cerebrospinal fluid but not serum levels of APRIL and BAFF

BAFF and APRIL in CSF and serum were analysed for covariance. Levels of APRIL and BAFF in CSF showed a weak correlation (Spearman *r *= 0.27, *P *< 0.01; Figure 3c). No correlation between APRIL and BAFF could be detected in serum (Figure 3d).

### No covariation between interleukin-6 and BAFF or APRIL levels in systemic lupus erythematosus patients

It has been reported that the inflammatory cytokine IL-6 is raised in the CSF of NPSLE patients compared with SLE patients without CNS symptoms [40], and this could be confirmed also in the present CSF samples (49 ± 68 pg/ml in NPSLE patients versus 26 ± 44 in SLE patients without CNS symptoms; *P *< 0.05). In an attempt to determine whether BAFF and APRIL are produced independently of IL-6, we analysed APRIL and BAFF, respectively, for covariance with IL-6. No correlation was observed between IL-6 and APRIL levels (Figure 3e) or between IL-6 and BAFF levels (Figure 3f).

## Discussion

To our knowledge this study is the first to show that SLE patients have elevated levels of APRIL and BAFF in CSF. SLE patients displayed levels of APRIL in CSF that were 24-fold higher than those of healthy controls, and levels of BAFF that were 200-fold higher, thus suggesting high intrathecal levels of APRIL and BAFF to be a feature of SLE. Although increased levels of both BAFF and APRIL have been reported earlier [27,41], these cytokines were measured in serum, where the differences between SLE patients and healthy controls were more modest. Due to the greater strictness of the NPSLE criteria in comparison with the 1987 American College of Rheumatology Case definitions for SLE [35] that we have previously used [10] and that we also employed in this study, patients with mild NPSLE could potentially be grouped with SLE patients without CNS inflammation. Nevertheless, we were able to find significant differences in APRIL levels but not in BAFF levels between SLE patients and NPSLE patients.

Mechanisms associated with the pathogenesis of NPSLE include anti-neuronal antibodies, anti-phospholipid antibody-associated thrombosis, and (rarely) vasculitis by immune complex depositions. Thus, pathogenic antibody formation is strongly associated with the manifestations of NPSLE. We found elevated levels of the antibody-inducing cytokine APRIL in the CNS of NPSLE patients compared with SLE patients without CNS involvement. Elevated levels of IL-6 in CSF have been reported in NPSLE patients [40] and could be confirmed in the present patient material. Since BAFF, APRIL, and IL-6 are important players in the survival, differentiation, and isotype switching of B cells, they may have an important role in the aetiology of NPSLE.

Analyses of covariance revealed no correlation between serum and CSF levels of BAFF or APRIL, suggesting that APRIL and BAFF in CSF are produced locally instead of systemically and subsequently passed to the CSF. A potential source of APRIL and BAFF in the CNS are the astrocytes of the brain. In MS patients, astrocytes have been established as producers of both BAFF and APRIL. APRIL in the CNS was expressed only by reactive astrocytes and increased in MS lesions [42]. Similarly, the transcript level of BAFF was 10-fold higher in MS lesions compared with normal CNS [43]. Collectively, this suggests that astrocytes do not constitutively express high levels of APRIL and BAFF, but rather upregulate the expression of these cytokines upon inflammation. BAFF and APRIL are expressed by a number of other cell types, including monocytes, macrophages, dendritic cells (DCs), neutrophils, and T cells [44]. Although the CNS was initially considered an immunologically privileged site, one of the primary events that occur during CNS affecting autoimmune disease such as MS is the recruitment of immunocompetent lymphocytes to the brain [45]. In lupus-prone MRL-Ipr mice, T cells pass the blood-brain barrier to infiltrate the brain [46,47]. Even though T-cell infiltration in the CNS of SLE patients has not been firmly established, T cells cannot be ruled out as a source of intrathecal BAFF and APRIL in SLE patients.

Analyses of covariance between APRIL and BAFF levels in the CSF of SLE patients suggested that BAFF and APRIL covary and thus are regulated together. IFN-α, IFN-γ, IL-10, and CD40L are important in the upregulation of both BAFF and APRIL [48,49]. SLE patients have large increases in the levels of IFN-α, IL-10, and soluble CD40L. Furthermore, T and B cells from SLE patients express highly increased levels of CD40L [50-52]. IFN-α, IL-10, and CD40L therefore could account for the upregulation of BAFF and APRIL seen in SLE patients. How BAFF and APRIL production in the CNS is regulated is currently not known but warrants further investigation.

SLE has been described as a disease of B-cell hyperactivity [53]. A number of studies concur in showing that DCs play a major role in B-cell development, mostly through the production of cytokines such as BAFF, IL-12, IL-6, and IFN-α [54]. CD40L treatment of bone-marrow-derived DCs from lupus-prone B6.TC mice induced higher production of IL-6, IL-10, and TNF-α than in B6 mice [55]. In an attempt to investigate whether IL-6, BAFF, and APRIL are regulated together, perhaps through the involvement of DCs, we analysed covariance between IL-6 and APRIL or BAFF. Although IL-6 levels were increased in the CSF of NPSLE patients, we could not correlate IL-6 to APRIL or BAFF levels. IL-6 is not known to induce BAFF and APRIL. In the development of humoral immunity, BAFF particularly supports the survival of immature transitional and mature B cells [24]. IL-6, on the other hand, promotes the transition of plasmablasts to plasma cells [56]. BAFF, APRIL, and IL-6 therefore likely have complementary roles in the autoantibody formation seen in SLE.

BAFF and APRIL are known to form homotrimers [57,58]. However, it has been shown that BAFF and APRIL can associate with each other to form a heterotrimer capable of stimulating B-cell proliferation. The levels of heterotrimers of BAFF/APRIL are nondetectable in the serum of healthy controls, whereas they are increased in patients with autoimmune diseases such as SLE [59]. We do not know whether the antibodies used in our BAFF- and APRIL-specific ELISAs recognise only monomeric and homotrimeric molecules of BAFF and APRIL or whether they are also able to recognise heterotrimers of BAFF/APRIL. If CSF levels of BAFF/APRIL heterotrimers are increased in the CSF similarly to the levels seen in serum, and the BAFF- and APRIL-specific antibodies used in our study are unable to recognise heterotrimers, we may have underestimated the levels of BAFF and APRIL in CSF. Regardless of this possibility, we did find elevated levels of BAFF and APRIL in the CSF of SLE patients.

Evidence suggests a major contribution of B cells to the pathogenesis of NPSLE, an effect supported by the salutary effect of the B-cell-depleting monoclonal antibody rituximab in this condition [60]. BAFF receptors and CD20 overlap in many B-cell subsets, but they differ in certain populations such as plasma cells, which express BCMA but not CD20. Blockage of BAFF therefore might provide distinct advantages over B-cell depletion therapy that targets CD20 (that is, rituximab). Another concern is that rituximab treatment as a side effect causes elevated BAFF levels in serum [61]. Therefore, upon cessation of treatment, newly generated immature cells could be exposed to high levels of BAFF, causing a resurgence of autoimmunity. This, in theory, could be averted by targeting BAFF. The potentially beneficial effects of BAFF and/or APRIL blockage are underscored in experimental autoimmune encephalomyelitis (a mouse model of MS). Besides demyelating disease, these mice have elevated immunoglobulin levels and spontaneously secrete autoantibodies to DNA, which eventually leads to autoimmune manifestations similar to SLE. Treatment with a BAFF/APRIL antagonist (soluble BCMA-Fc) is able to inhibit these autoimmune manifestations [62].

Since BAFF and APRIL have a major impact on B-cell survival, T-cell function, and antibody production [11,12], overexpression of these cytokines could contribute to increased lymphocyte survival and proliferation, potentially leading to increased leucocyte infiltration into the CNS. Moreover, upregulation of BAFF expression *in vivo *can result in the rescue of self-reactive B cells from elimination [63]. Collectively, these findings suggest that treatment with BAFF and/or APRIL antagonists could be beneficial in SLE treatment. A phase II study in rheumatoid arthritis and SLE patients using LymphoStat-B, a fully humanised antibody specific for human BAFF (also known as Belimumab), showed modest efficacy in rheumatoid arthritis [64], while a phase II study in SLE patients did not meet the primary efficacy endpoints [65]. The decoy receptor TACI-Ig (Atacicept) preventing the binding of BAFF and APRIL to the receptor TACI on B cells led to improvements in animal models of lupus [66] and arthritis [67]. Its clinical value is currently under investigation in rheumatoid arthritis and SLE.

## Conclusion

We have found elevated levels of APRIL and BAFF in the CSF of SLE patients. APRIL was augmented in NPSLE patients compared with SLE patients without CNS involvement. Potential implications of our findings could be that APRIL and BAFF measurements in the CSF could aid in the diagnosis of SLE. Furthermore, APRIL and BAFF antagonists breeching the blood-brain barrier could have beneficial effects on SLE patients, in particular patients with NPSLE.

## Abbreviations

APRIL: a proliferation-inducing ligand; ARA: American Rheumatism Association; BAFF: B-cell activating factor of tumour necrosis factor family; BCMA: B-cell maturation antigen; CNS: central nervous system; CSF: cerebrospinal fluid; DC: dendritic cell; ELISA: enzyme-linked immunosorbent assay; GFP: glial fibrillary acidic protein; IFN: interferon; IL: interleukin; MRI: magnetic resonance imaging; MS: multiple sclerosis; NFL: neurofilament; NPSLE: neuropsychiatric systemic lupus erythematosus; SD: standard deviation; SLE: systemic lupus erythematosus; TACI: transmembrane activator and calcium-modulating cyclophilin ligand interactor; TNF: tumour necrosis factor.

## Competing interests

The authors declare that they have no competing interests.

## Authors' contributions

KE designed the study, was involved in the management of the study, and helped to interpret the results. AG-C was involved in the management of the study, performed the laboratory investigations, analysed the data statistically, helped to interpret the results, and prepared the manuscript. ET was involved in the management of the study, collected the clinical data, and helped to interpret the results. AG-C and ET contributed equally to this work. All authors read and approved the final manuscript.
